# Altered Cellular Immunity and Differentially Expressed Immune-Related Genes in Patients With Systemic Sclerosis–Associated Pulmonary Arterial Hypertension

**DOI:** 10.3389/fimmu.2022.868983

**Published:** 2022-05-18

**Authors:** Jianxin Tu, Jinji Jin, Xiaowei Chen, Li Sun, Zhen Cai

**Affiliations:** ^1^ Bone Marrow Transplantation Center, Department of Hematology, The First Affiliated Hospital, College of Medicine, Zhejiang University, Hangzhou, China; ^2^ Department of Rheumatology, The First Affiliated Hospital of Wenzhou University, Wenzhou, China; ^3^ Gastrointestinal Surgery, The First Affiliated Hospital of Wenzhou University, Wenzhou, China

**Keywords:** systemic sclerosis, pulmonary arterial hypertension, cellular immunity, IL-7R, LCK, HDAC1, early prediction

## Abstract

Systemic sclerosis (SSc) is the most common connective tissue disease causing pulmonary hypertension (PAH). However, the cause and potential immune molecular events associated with PAH are still unclear. Therefore, it is particularly essential to analyze the changes in SSc-PAH–related immune cells and their immune-related genes. Three microarray datasets (GSE22356, GSE33463, and GSE19617) were obtained by the Gene Expression Omnibus (GEO). Compared with SSc, we found neutrophils have a statistically higher abundance, while T-cell CD4 naive and T-cell CD4 memory resting have a statistically lower abundance in peripheral blood mononuclear cells (PBMCs). Moreover, the results of Gene Set Enrichment Analysis (GSEA) showed there is a differential enrichment of multiple pathways between SSc and SSc-PAH. By combining differentiated expressed genes (DEGs) and immune-related genes (IRGs), fifteen IRGs were selected. In addition, we also analyzed the first five rich Kyoto Encyclopedia of Genes and Genomes (KEGG) pathways and the most abundant Gene Ontology (GO)-molecular functional terms. Furthermore, interleukin-7 receptor (IL-7R), tyrosine–protein kinase (LCK), histone deacetylase 1 (HDAC1), and epidermal growth factor receptor (EGFR) genes were identified as hub genes *via* protein–protein interaction (PPI) network analysis. The Comparative Toxic Genomics Database (CTD) analysis result showed that LCK, HDAC1, and EGFR have a higher score with SSc. Coexpression network analysis confirmed that IL-7R, LCK, and HDAC1 are key genes related to immune regulation in SSc without PAH and are involved in T-cell immune regulation. Subsequently, using GSE22356 and GSE33463 as the test sets and GSE19617 as the verification set, it was verified that the mRNA expression levels of the three central genes of SSc-PAH were significantly lower than those of the SSc without PAH samples. Consistent with previous predictions, the expressions of IL-7R, LCK, and HDAC1 are positively correlated with the numbers of T-cell CD4 naive and T-cell CD4 memory, while the expressions of IL-7R and LCK are negatively correlated with the numbers of neutrophils in the peripheral blood. Therefore, this evidence may suggest that these three immune-related genes: IL-7R, LCK, and HDAC1, may be highly related to the immunological changes in SSc-PAH. These three molecules can reduce T cells in SSc-PAH PBMCs through the regulation of T-cell activation, which suggests that these three molecules may be involved in the development of SSc-PAH. Meanwhile, the low expression of IL-7R, LCK, and HDAC1 detected in the peripheral blood of SSc may indicate the possibility of PAH and hopefully become a biomarker for the early detection of SSc-PAH. Finally, 49 target miRNAs of 3 specifically expressed hub genes were obtained, and 49 mRNA–miRNA pairs were identified, which provided directions for our further research.

## Introduction

Systemic sclerosis (SSc) is a complex autoimmune connective tissue disease that affects almost all major organs of the human body and is the rheumatic immune disease with the highest mortality rate (1). SSc is characterized by progressive vascular disease, accompanied by vascular remodeling such as hardened finger skin, pulmonary hypertension (PAH), and abnormal microcirculation (2). Vascular disease is a common symptom in patients with systemic sclerosis and is often the earliest manifestation of the disease (1, 2).

PAH is a devastating disease that can cause severe disability and often leads to death (3). Connective tissue disease is a common cause of pulmonary hypertension (4). Among them, SSc is the most common connective tissue disease with PAH (5). About 8%–12% of SSc can be combined with PAH (6), accounting for almost 75% of connective tissue disease-related PAH cases (7). In recent years, with the improvement of the treatment of scleroderma renal crisis, the survival rate of SSc has been significantly improved, but the mortality rate of SSc-PAH is still very high (8). The survival rate of SSc-PAH is much lower than that of SSc patients without PAH, and their 3-year survival rates are 56% vs. 94%, respectively (9). PAH has been the primary complication of death in SSc in recent years (9, 10).

Recently, accumulating evidence from preclinical and clinical studies has highlighted the role of inflammation in the development of PAH disease. For example, some inflammatory conditions, such as connective tissue disease, are associated with an increased incidence of PAH. In addition in lung biopsies of PAH patients, nearly all inflammatory cell lineages were located near the remodeled pulmonary vessels, mainly composed of macrophages, mast cells, T lymphocytes, B lymphocytes, dendritic cells, and neutrophils (11).

At present, the pathogenesis of PAH in systemic sclerosis is still unclear. Immune abnormalities, including autoimmune abnormalities and immune cell infiltration and activation, are the key features of systemic sclerosis (12). Most studies have shown that vascular injury is an activator of immune cells (13–15). More and more evidence indicates that abnormal immunity is considered to be a critical part in the development of SSc-PAH (16, 17). Immune abnormalities could not only promote the occurrence of fibrosis in SSc (12) but also relate to SSc vascular disease (13). However, how immune abnormalities connect to the vasculopathy and fibrosis in SSc is still poorly understood.

Therefore, it is a very meaningful study to explore the immune abnormalities and differentially expressed immune-related genes in SSc-PAH and SSc patients. It can help us better understand the immune changes that occur in the secondary PAH process of SSc, reveal the pathogenesis of PAH in SSc, and provide the foundation for the early prediction and immunotherapy of SSc-PAH.

However, most of the above evidence mainly comes from serological or pathological studies in patients or animal models, and the crucial genes responsible for immune abnormalities in SSc-PAH remain unclear. Due to the rapid development of gene chip technology, researchers could quickly detect the gene expression differences in a disease, helping researchers to better understand the pathogenesis of the disease at the genetic level.

Therefore, in this study, we used gene expression data from the Gene Expression Omnibus (GEO) (http://www.ncbi.nlm.nih.gov/geo/), which was published and identified cellular immunity changes between SSc-PAH and SSc without PAH groups. Furthermore, we also comprehensively analyzed the hub immune-related genes (IRGs) that may cause immune abnormalities between the two groups, as well as the involved pathways and regulatory mechanisms, and further explored the miRNA regulatory network related to these genes. Our research revealed the abnormal immune cell changes in PBMCs of SSc-PAH and differentially expressed IRGs in SSc patients with PAH, providing the foundation for the early prediction and immunotherapy of SSc-PAH.

## Method

### Microarray Data Acquisition

Microarray datasets were selected from the GEO (http://www.ncbi.nlm.nih.gov/geo). Selecting criteria included the following: (1) *Homo sapiens* expression profiling by array; (2) peripheral blood mononuclear cells (PBMs) in scleroderma patients with and without pulmonary hypertension; (3) datasets containing more than ten samples; and (4) datasets containing complete information about the samples. Based on these criteria, the GSE22356, GSE33463, and GSE19617 datasets were obtained. GSE22356 and GSE33463 were selected as test sets, and GSE19617 was selected as the validation sets. GSE22356 contained 10 scleroderma patients with pulmonary hypertension and 10 scleroderma patients without pulmonary hypertension. GSE33463 contained 50 scleroderma patients with pulmonary hypertension and 19 scleroderma patients without pulmonary hypertension. GSE19617 contained 15 scleroderma patients with pulmonary hypertension and 21 scleroderma patients without pulmonary hypertension.

### Data Preprocessing

Series matrix files were converted from the gene probe IDs to gene symbol codes. After merging GSE22356 and GSE33463 microarray data, batch effects were regulated by the “combat” function of “sva” package of R software, utilizing empirical Bayes frameworks. The final step is to normalize the expression values through the “Limma” package in the R software so that the expression values have a similar distribution across a set of arrays.

### CIBERSORT Analysis Immune Cell Change

In this study, Cell Type Identification By Estimating Relative Subsets Of RNA Transcripts (CIBERSORT) was used to analyze the normalized data filtered by the Perl programming language to obtain an immune cell change. The study included 22 immune cells. These immune cells are B-cell naive, B-cell memory, plasma cell, T-cell CD8, T-cell CD4 naive, T-cell CD4 memory activation, T-cell CD4 memory resting, T-cell regulatory (Tregs), T-cell gamma delta, NK cell resting, NK cell activated, monocytes, macrophage M0, macrophage M1, macrophage M2, dendritic cell resting, dendritic cell activated, mast cell resting, mast cell activated, eosinophils, and neutrophils. The relationship between two immune cells and the percentage of immune cells in the gene expression matrix were calculated by installing the “corrplot” package, and then the data of different groups were plotted using a vioplot.

### Differentially Expressed Genes, Enrichment Analysis, and Gene Set Enrichment Analysis

Differentially expressed genes (DEGs) between SSc and SSc-PAH samples were identified by the R package “Limma.” An adjusted *p*-value <0.05 and |log2fold change| ≥1 were used as cutoff values. Differentially expressed IRGs are the intersection between IRGs and DEGs. There are a total of 1,901 IRGs downloaded from the Immport website (www.immport.org), which is funded by the National Institutes of Health (NIH), National Institute of Allergy and Infectious Disease (NIAID), and Division of Allergy, Immunology and Transplantation (DAIT). Gene Ontology (GO) enrichment and Kyoto Encyclopedia of Genes and Genomes (KEGG) were then analyzed by the R package “cluster Profiler.” *p* < 0.05 was considered statistically significant. Gene Set Enrichment Analysis (GSEA) v4.1.0 software was used to identify immunological features in SSc and SSc-PAH groups. The number of permutations was set at 1,000 for each analysis. Additionally, to sort the enriched pathways in each phenotype, we utilized the nominal *p*-value and normalized enrichment score (NES), which were considered to be significantly enriched.

### Protein–Protein Interaction Network Construction

To construct an interactive network of overlapping DEGs, the Search Tool for the Retrieval of Interacting Genes (STRING, http://string-db.org) (18) was well utilized. Subsequently, the results were visualized using Cytoscape. In this network, the significant genes were identified as hub genes by CytoHubba (19).

### Weighted Coexpression Network Construction

The WGCNA software package was used to construct gene coexpression networks. In the first step, outliers in the gene expression matrix were filtered by hierarchical clustering analysis. In the second step, the correlation coefficients of genes are constructed and converted into a weighted adjacency matrix. In the third step, these genes were assigned to the smallest size modules and a cluster dendrogram was drawn, which was then combined with a height cutoff (cutoff <0.25). Finally, modules significantly associated with groups were selected, and their biological functions were explored by GO and KEGG analyses.

### Potential Crucial Gene Identification and Candidate miRNA Prediction

In this study, we used data from the Comparative Toxic Genomics Database (CTD, http://ctdbase.org/) to analyze the association between potential key genes and SSc risk. The CTD is an innovative digital ecosystem that assists us in synthesizing information (including chemical-gene/protein interactions, chemical-disease, and gene-disease relationships) to develop hypotheses related to disease mechanisms (20).

Upstream binding miRNAs of hub genes were selected by five target gene prediction programs, including Target Scan (http://www.targetscan.org/vert_71/), mirDIP (http://ophid.utoronto.ca/mirDIP/), miRDB (http://mirdb.org/), DIANA [DIANA tools-Tarbase v8 (athena-innovation.gr)], miRmap [miRmap (ezlab.org)]. And we used Venn, an interactive Venn diagram viewer, to conduct an intersection analysis.

### Statistical Analysis

IBM SPSS Statistics 23 was utilized to analyze the data and illustrate the receiver operating characteristic (ROC) curve. The Spearman’s correlation coefficient was utilized to assess the association between continuous variables. A *t*-test was utilized to compare the differences between the two groups. Datesmerge, process, and analysis were conducted by R-3.5.3, and the ggplot2 package was utilized to illustrate box plots.

## Results

### The Changes of Immune Cells in PBMCs

**Figure 1:**
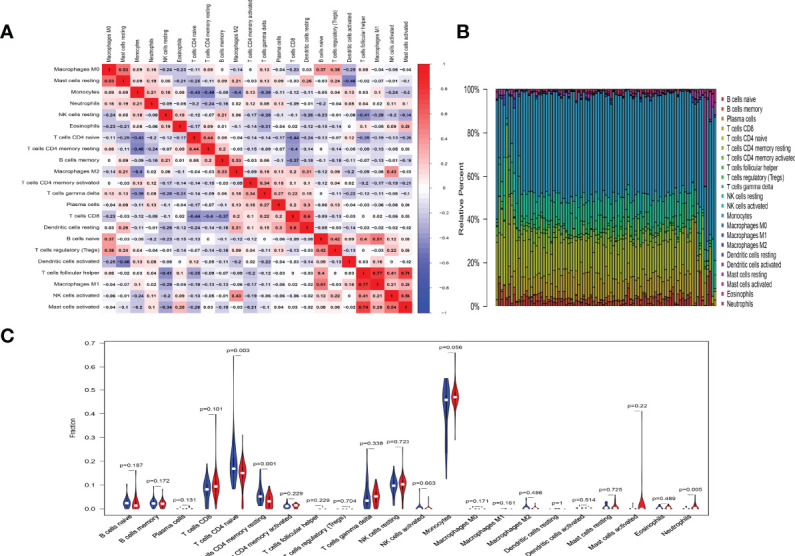
Results of immune cells change. **(A)** Correlation matrix of the changes in number of 22 immune cells in SSc PBMCs. Red indicates a positive correlation; blue indicates a negative correlation. The larger the absolute value of the number, the more positive or negative correlation there is. **(B)** Landscape of immune cells changes. **(C)** A violin diagram of the immune cell proportions in two groups. The blue fusiform fractions on the left represent the SSc group, and the red fusiform fractions on the right represent the SSc-PAH group.

In order to research the changes of immune cells in the peripheral blood of SSc and SSc-PAH patients, peripheral blood cell (PBMC) microarray data from the GSE22356 and GSE33463 were analyzed, which included 29 SSc samples and 60 SSc-PAH samples. We used an established computational resource (CIBERSORT) to explore gene expression profiles of GEO-downloaded samples to infer the density of 22 types of immune cells. The corheatmap **(** **Figure 1A** **)** result showed that macrophage M1 and T-cell follicular helpers had a significant positive correlation (*R* = 0.77). T-cell CD4 memory resting had a negative correlation with monocytes (*R* = −0.48). The heatmap of the correlation analysis (**Supplementary Figure S1**) summarizes the results obtained from the 69 filtered gene expression matrices, and **Figure 1B** shows the relative percentages of the 22 immune cells. Compared with the SSc group, the violin plot of the immune cells showed that T-cell CD4 naive and T-cell CD4 memory resting have statistically lower abundance, while neutrophils have statistically higher abundance in PBMCs (**Figure 1C** **).**

### Enrichment Analysis

The pathways involved in the two expression datasets were analyzed by GSEA and showed significant differences in enrichment from the KEGG Collection (*p*-value <0.05). The results of the GSEA analysis showed that ribosome, spliceosome, RNA degradation, RNA polymerase, and basal transcription factor pathways showed significantly differential enrichment in the SSc group, and neuroactive ligand receptor interaction, complement and coagulation cascades, systemic lupus erythematosus, linoleic acid metabolism, and arachidonic acid metabolism pathways showed significantly differential enrichment in the SSc-PAH group (**Supplementary Figure S2**; **Table 1**).

**Table 1:** The significantly differential enrichment pathways between the SSc without PAH and SSc-PAH groups.

Gene set name	NES	NOM *p*-value
KEGG_RIBOSOME	1.65	0.046
KEGG_SPLICEOSOME	1.53	0.002
KEGG_RNA_DEGRADATION	1.48	0.006
KEGG_RNA_POLYMERASE	1.43	0.04
KEGG_BASAL_TRANSCRIPTION_FACTORS	1.42	0.036
KEGG_NEUROACTIVE_LIGAND_RECEPTOR_INTERACTION	−1.88	0
KEGG_COMPLEMENT_AND_COAGULATION_CASCADES	−1.85	0
KEGG_SYSTEMIC_LUPUS_ERYTHEMATOSUS	−1.63	0.013
KEGG_LINOLEIC_ACID_METABOLISM	−1.61	0.037
KEGG_ARACHIDONIC_ACID_METABOLISM	−1.47	0.044

### Identification of Differentially Expressed Genes

**Figure 2:**
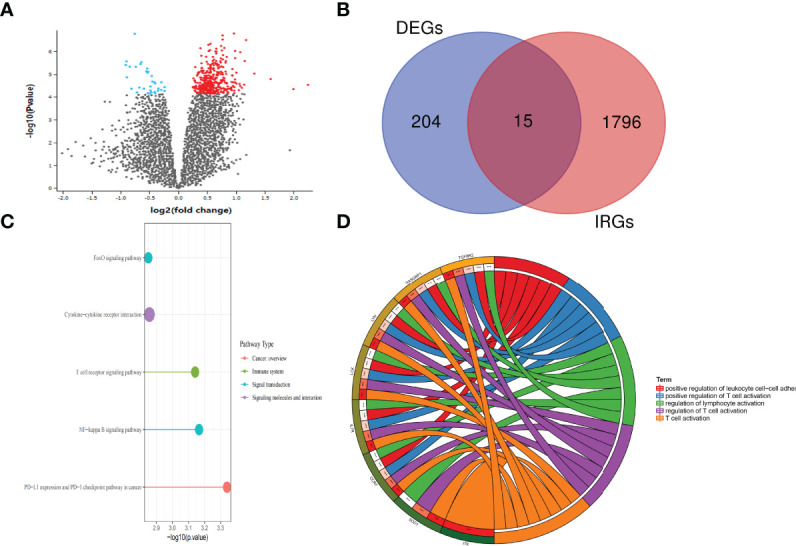
Differential expression of genes between the SSc and SSc-PAH groups. **(A)** Volcano plot of the differentially expressed genes between SSc and SSc-PAH. Black points represent the adjusted *p*-value >0.05. Blue points represent adjusted *p*-value <0.05 and downregulated genes. Red points represent adjusted *p*-value <0.05 and the upregulated genes. **(B)** The Venn diagram of differentially expressed IRG intersection between IRGs (1,901 IRGs downloaded from the Immport website) and DEGs (219 DEGs selected). **(C)** Enrich KEGG class plot reveals the top 5 IRGs. The top 5 most enriched KEGG pathways of those 15 IRGs were as follows: PD-L1 expression and PD-1 checkpoint pathway in cancer, NF-kappa B signaling pathway, T-cell receptor signaling pathway, and FoxO signaling pathway. **(D)** Chord plot shows the top 5 most enriched Gene Ontology (GO)-molecular function terms: T-cell activation, regulation of T-cell activation, regulation of T-cell activation, positive regulation of T-cell activation, and positive regulation of leukocyte cell–cell adhesion. ***p-value < 0.001.

DEGs in two group patients were analyzed using the “Limma” package. With the cutoff value of |log2 (fold change) |> 1 and adjusted *p* < 0.05, 182 significantly upregulated genes and 37 significantly downregulated genes were identified **(** **Figure 2A** **;** **Table 2** **)**. There are a total of 1,901 IRGs downloaded from the Immport website (www.immport.org), which is funded by the NIH, NIAID, and DAIT. Based on the list of differentially expressed genes, 15 differentially expressed IRGs were selected **(** **Figure 2B** **).** The top 5 most enriched KEGG pathways of those 15 IRGs were as follows: PD-L1 expression and PD-1 checkpoint pathway in cancer, NF-kappa B signaling pathway, T-cell receptor signaling pathway, and FoxO signaling pathway **(** **Figure 2C** **)**. The top 5 most enriched GO-molecular function terms were T-cell activation, regulation of T-cell activation, regulation of T-cell activation, positive regulation of T-cell activation, and positive regulation of leukocyte cell–cell adhesion **(** **Figure 2D** **)**. The top 5 most enriched GO-cellular component and molecular function terms are shown in **Supplementary Figure S3**.

**Table 2:** The 182 significantly upregulated genes and 37 significantly downregulated genes that were identified between the SSc and SSc-PAH groups.

**Upregulated genes**	ING5 NOB1 RPS23 HP1BP3 AQP3 CEP68 ZCCHC4 EEF1B2 RPS3 ZNF121 SCML4 RPL7 RPL17 ZBTB41 CAMK4 RPL3 RPL27A COMMD6 RPL35A NELL2 ZNF573 HERC2P2 EIF4A2 NOG RPL10A CREBL2 INTS6L POP5 FBL RAPGEF6 RPS13 RPL31 IL7R CCNL2 SNORD32A LEF1 RPS10 DNAJC16 CASP8AP2 LTB CCR7 ZFP62 RPS4X SPTBN1 POGZ FBXL16 TRAF3IP3 NKHD1-EIF4EBP3 EIF3H MRPL57 NR3C2 DGKA RPL23A XIST RRS1 RPS21 C16orf54 RPL37 PDE7A RPL10 EIF3E RPSA TRIM74 RBL2 RPL24 OXNAD1 CD27 RPS20 RIPOR2 TOP2B AK5 AGL MAN1C1 LDHB COX7C LNPEP ZNF137P NAA16 ALMS1 RPS16 KPNA5 SLC26A11 RPS6 ELK4 TOMM7 TBCA RPL13A WDR82 NPM1 SLC38A1 RPLP0 SATB1 PTCD3 SNORD42A CHMP7 PATJ TARDBP PJA1 SRSF10 SOD1 ZRANB2 CEP120 TXLNG ZNF814 CHCHD6 ETS1 BCL11B PIK3IP1 EPHX2 TBC1D4 DNMT3A TCF7 NOLC1 LCK HDAC1 CDC14A PRAG1 USP45 RPS29 UXT ATP8B2 RPL22 HINT1 OGT ITK JADE1 CCT4 DOCK9 TNRC6C ZNF776 RPS14 EEF1D BRMS1L ANAPC16 BACH2 RPS15A SUCO C12orf57 RPL27 PCNX2 RPL30 CTCF PHC3 RGPD5 IMPDH2 TGFBR2 ERCC5 RETREG1 CETN3 MAL ATM HSPD1 RPL34 SON SLTM RPL9 TUT1 EDAR N4BP2L2 PRKACB LRRN3 EBAG9 RASGRP1 RCAN3 NARS2 ZC3H13 CCNB1IP1 KIAA1147 MYO9A LIX1L AMMECR1 ATR NLRC3 ADH5 RPS27A DNAJC19 ZNHIT3 ARHGEF18 NAE1 GIMAP2 AURKAP1 OXCT1
**Downregulated genes**	JSRP1 HBQ1 TIMP1 CYP2A13 HIST1H2BC ITPKA MISP3 ARHGEF12 SMIM1 LSMEM1 SAT1 RELT THY1 EGFR LEPROT KLK3 OSBP2 E2F2 GAST NT5M LYN RAB31 LILRB2 PSAP GNAO1 SPINK4 SPATA46 ANKRD9 MKI67 SMOX GM2A LY6G6D GATA1 SERPINA1 CA5A GCGR KLHDC8B

### Protein–Protein Interaction Network Analysis and Hub Gene Identification

**Figure 3:**
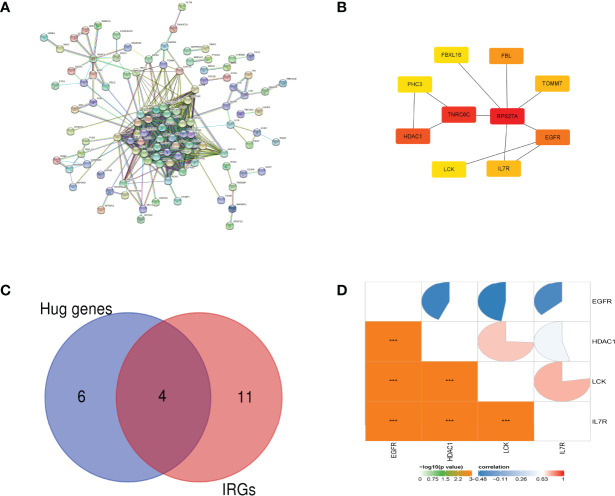
PPI network analysis and hub gene identification. **(A)** The interaction network between proteins coded by DEGs. **(B)** Ten hub genes were screened by the network recognition ensemble algorithm. **(C)** The Venn diagram of differentially expressed IRG intersection between IRGs and hub genes. **(D)** The pie chart of the Pearson correlation analysis shows the correlation between 4 IRGs. ^***^ *p*-value < 0.001.

The interaction network between proteins coded by DEGs, which was composed of 214 nodes and 776 edges, was constructed by STRING and visualized by Cytoscape **(** **Figure 3A** **)**. Next, we used the CytoHubba plugin to identify hub genes. The network recognition ensemble algorithm is a bottleneck algorithm in Cyctoscape. We use bottleneck algorithm of the CytoHubba plugin in Cytoscape to screen 10 hub genes. Ten hub genes were selected by network recognition ensemble algorithm, including TOMM7, epidermal growth factor receptor (EGFR), interleukin-7 receptor (IL-7R), tyrosine–protein kinase (LCK), RPS27A, TNRC6C, histone deacetylase 1 (HDAC1), PHC3, FBXL16, and FBL **(** **Figure 3B** **)**. Ten hub genes were screened when using the bottleneck algorithm. Only IL-7R, LCK, HDAC1, and EGFR were identified by interacting with the 15 IRGs **(** **Figure 3C** **)**. We further investigated the correlation between the four genes. The results showed that the expressions of IL-7R, LCK, and HDAC1 genes are positively correlated but negatively correlated with the expressions of EGFR **(** **Figure 3D** **).**

### Identification of Potential Crucial Genes Associated With SSc

**Figure 4:**
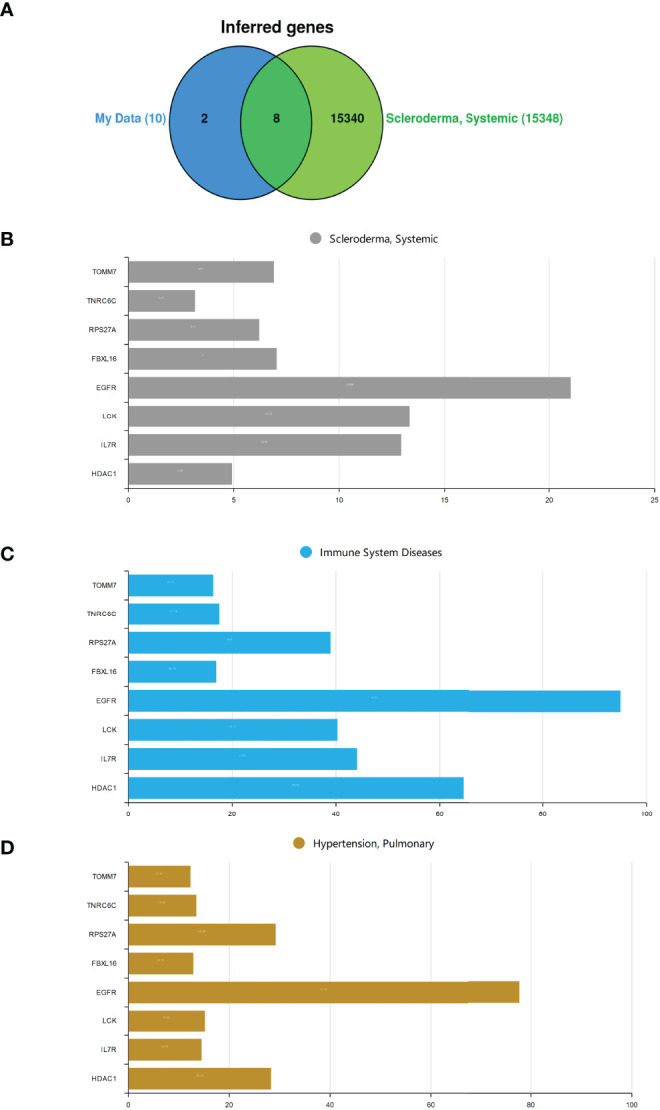
|The hub genes analysis in CTD. **(A)** The Venn diagram of intersection between hub genes and SSc crucial genes. **(B)** Systemic sclerosis. **(C)** Immune system diseases. **(D)** Pulmonary hypertension.

CTD was employed to research the interaction between SSc and potential crucial genes. **Figure 4A** shows that eight of the previously predicted ten hub genes could be inferred to be associated with SSc. We also explore those potentially crucial genes that target immune system diseases and pulmonary hypertension. Inference scores in CTD indicated the association between disease and genes. The interaction results revealed that EGFR, LCK, and IL-7R have a higher score for SSc **(** **Figure 4B** **)**. EGFR and HDAC1 have a higher score for immune system diseases **(** **Figure 4C** **)**. EGFR and HDCA1 have a higher score for pulmonary hypertension **(** **Figure 4D** **).** It is worth mentioning that EGFR has high scores in all groups **(** **Figure 4** **).**

### Crucial Modules Identification *via* WGCNA

**Figure 5:**
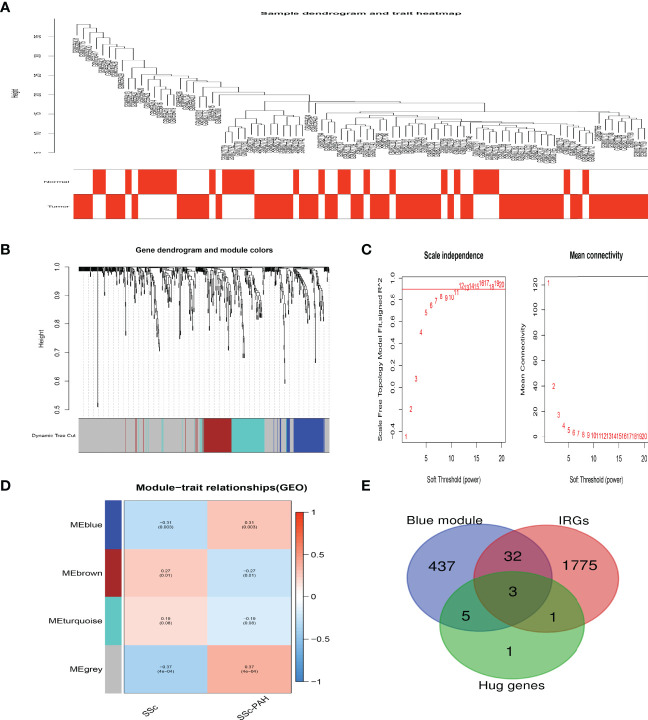
Crucial modules in SSc-PAH. **(A)** Sample dendrogram and trait heat map. **(B)** Clustering dendrograms of genes based on a dissimilarity measure (1-TOM). **(C)** Analysis of the scale-free fit index (left) and the mean connectivity (right) for various soft-thresholding powers. **(D)** Module-trait associations were evaluated by correlations between module eigengenes and sample traits. **(E)** The Venn diagram indicating 3 genes from the blue module, ten hub genes, and IRGs.

In order to screen the crucial genes related to the immunomodulation in SSc and SSc-PAH, WGCNA was utilized to perform the coexpression network analysis. Four modules were identified in this study **(** **Figures 5A–D** **).** The association between the modules and groups was assessed by the correlation between module eigengene (ME) values and groups. Heatmap profiles were drawn to visualize the data. As shown in **Figure 5D**, we found three significant modules: grey, blue, and brown modules. After each module pathway enrichment analysis **(** **Supplementary Figure S4** **)**, the genes in the blue module were involved in T-cell immune regulation. Also, hub genes IL-7R, LCK, and HDAC1 were also in the blue module **(** **Figure 5E** **).**

### Identification of Hub Genes

**Figure 6:**
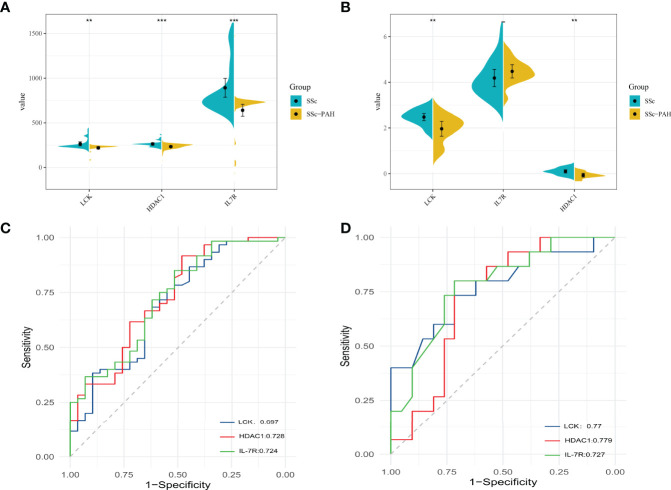
The mRNA expression levels of 3 hub genes between SSc and SSc-PAH. **(A)** The mRNA expression levels of 3 hub genes in test sets (GSE22356 and GSE33463). **(B)** The mRNA expression levels of 3 hub genes in validation sets (GSE19617). **(C)** The ROC curve of LCK levels, the HDAC1 levels, and the IL7R levels for predicting SSc without PAH diagnosis in test sets (GSE22356 and GSE33463). **(D)** The ROC curve of LCK levels, the HDAC1 levels, and the IL7R levels for predicting SSc without PAH diagnosis in validation sets (GSE19617). ^**^ *p*-value < 0.01; ^***^ *p*-value < 0.001.

To verify the expression differences of hub genes IL-7R, LCK, and HDAC1 between SSc and SSc-PAH, we used GSE22356 and GSE33463 as test sets and GSE19617 as the validation set, which included 21 SSc patients and 15 SSc-PAH patients. As shown in **Figures 6A, B**, compared with the SSc samples, the mRNA expression levels of the 3 hub genes in the SSc-PAH were significantly decreased (*p* < 0.05). To determine the significance of IL-7R, LCK, and HDAC1 in the diagnosis of SSc patient, ROC curve analyses were conducted to explore the sensitivity and specificity of crucial genes for SSc diagnosis. The ROC curve analysis of LCK (AUC: 0.697), HDAC1 (AUC: 0.728), and IL-7R (AUC: 0.724) **(** **Figure 6C** **)** for predicting SSc diagnosis was validated using GSE22356 and GSE33463. We then validated the **(** **Figure 6D** **)**, LCK (AUC: 0.77), HDAC1 (AUC: 0.779), and IL-7R (AUC: 0.727) using GSE19617. This indicates that the expression of IL-7R, LCK, and HDAC1 are related to the disease activity of SSc.

**Figure 7:**
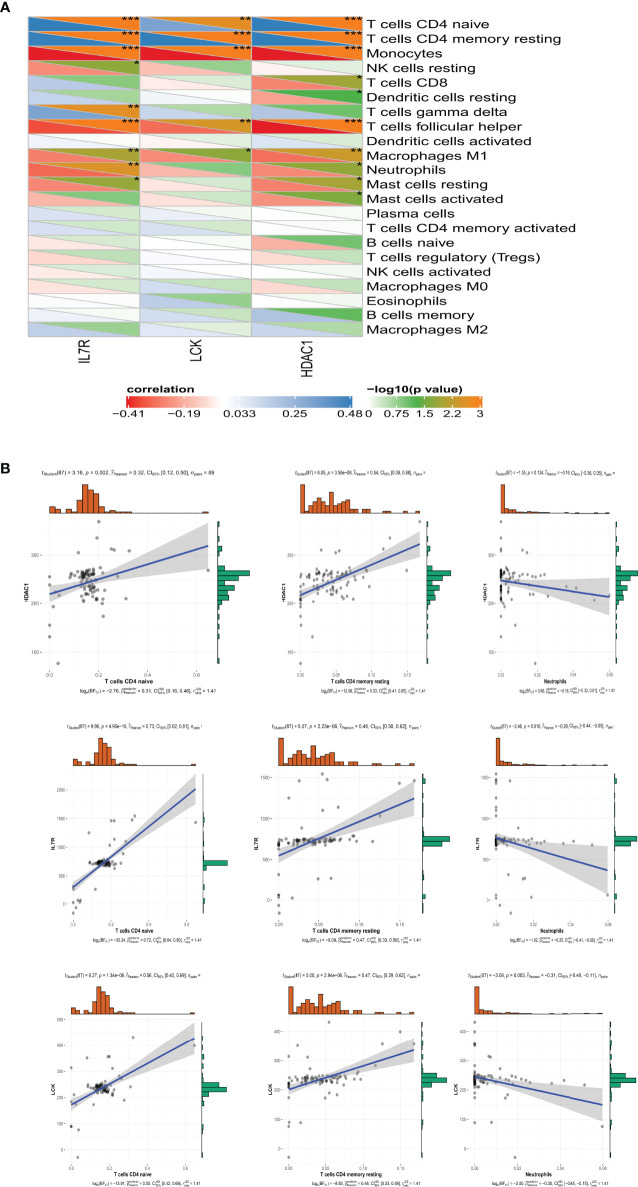
The relationship between hub genes and these immune cells. **(A)** The Pearson correlation analysis shows the correlation between 3 hub genes and these immune cells. ^*^ *p*-value < 0.05; ^**^ *p*-value < 0.01; ^***^ *p*-value < 0.001. **(B)** The relationship between IL-7R, LCK, an HDAC1 and T-cell CD4 naive, T-cell CD4 memory, and neutrophils.

According to the previous results, neutrophils have a higher abundance and T-cell CD4 naive and T-cell CD4 memory resting have lower abundance in SSc-PAH patient PBMC samples. We further explore the relationship between hub genes and these immune cells **(** **Figure 7A** **).** Consistent with our predictions, the expressions of IL-7R, LCK, and HDAC1 are positively correlated with the numbers of T-cell CD4 naiveness and T-cell CD4 memory; otherwise, the expressions of IL-7R, LCK, and HDAC1 are negatively correlated with the numbers of neutrophils in the peripheral blood **(** **Figure 7B** **).**

### Target miRNA Prediction and Coexpressed Network Construction

It has been demonstrated that microRNAs are responsible for the regulation of gene expression. Five online miRNA databases were utilized to predict target miRNAs of hub genes. Only the predicted miRNAs which commonly appeared in more than four programs, as mentioned above, were included for subsequent analysis. Lastly, 49 target miRNAs were selected from 4 specifically expressed hub genes and determined 49 mRNA–miRNA pairs **(** **Supplementary Figure S5** **)**.

## Discussion

Vasculopathy is an important feature of systemic sclerosis, leading to vasospasm, pulmonary artery remodeling, and microvascular occlusion (21). The exact cause of SSc-PAH is still unknown. The complex pathological features of SSc complicate the understanding of the function of the immune and vascular systems in the pathogenesis of SSc. Our imprecise understanding of the immune mechanisms that initiate SSc-PAH may make it difficult to identify effective treatments for the disease. Therefore, exploring the immune changes of SSc-PAH during the disease process and its possible key gene changes may provide new ideas for the treatment of SSc-PAH.

A series of complex interactions between inflammatory cells, vascular cells, and soluble mediators in the lung and periphery promote perivascular inflammation and—presumably—also pulmonary vascular remodeling in PAH. This includes: (1) inflammatory mediators and their effects on pulmonary vascular remodeling; (2) inflammatory/immune cells and their products in PAH; and (3) phenotypic changes in vascular cells and their feedback into the inflammatory and immune responses (11, 22). The fundamental role of the immune system in the development of PAH is becoming more widely recognized. Among them, the change in immune cells is the key link.

In this study, we found that in two groups of people with systemic sclerosis and systemic sclerosis with pulmonary hypertension, the difference in immune cells in PBMC analysis suggested that neutrophils have a statistically higher abundance, while T-cell CD4 naive and T-cell CD4 memory resting have a statistically lower abundance.

Many studies have shown that the adaptive immune system, with a large number of autoreactive T and B cells producing autoantibodies, plays a central role in the pathogenesis of SSc (12, 15, 16, 23, 24). Among them, T cells are present in SSc tissues and are considered to be the main factor driving endothelial dysfunction and fibrotic pathology in this disease, probably through the secretion of cytokines that ultimately promote macrophage, fibroblast, and myofibroblast activation of cells (25, 26).

In this study, we found that the numbers of T-cell CD4^+^ naive in PBMCs were significantly lower in abundance in SSc-PAH patients compared with SSc. While, T-cell CD4^+^ naive are activated after interaction with the antigen-MHC complex and differentiate into specific subtypes depending mainly on the cytokine milieu of the microenvironment. Besides the classical T-helper 1 and T-helper 2, other subsets have been identified, including T-helper 17, regulatory T cell, follicular helper T cell, and T-helper 9, each with a characteristic cytokine profile. The effector functions of these cells are mediated by the cytokines secreted by the differentiated cells (27). This may indicate specific immunomodulation in SSc-PAH tissue, resulting in a massive migration of T-cell CD4^+^ naïve into the tissue, thereby reducing the number of T cells in the peripheral blood. One of the limitations of this study is that immune infiltration changes in tissues were not obtained simultaneously. This is also an issue that needs to be explored in further research.

In SSc, many T cells, including Th1, Th2, Th17, Th22, T follicular helper (Tfh), Treg, and CD8^+^, are all involved in the pathogenic process (28–30).

Evidence has shown that CD8^+^ T cells and CD4^+^ CTL are the most prominent T cells in SSc lesions (28, 29). In addition to inducing apoptosis, CD4^+^ CTL secrete profibrotic and proinflammatory cytokines, including TGF-β and IL-1β. These cytokines may be directly involved in local tissue remodeling processes (31, 32). In addition, studies have confirmed that CD8^+^ T cells play an important role in fibrotic diseases. They can induce fibrosis through cytokines such as TNF-α and IL-13, and fibrosis can also lead to abnormal immune surveillance of CD8^+^ T cells (28). The current study indicated that a large number of apoptotic cells were found in the tissues of SSc patients, accompanied by an accumulation of CD8^+^ T cells and CD4^+^ CTL, indirectly suggesting that these cytotoxic T cells may target and kill the host cells (29). CD4^+^ CTL and CD8^+^ T cells in SSc may contribute to excessive tissue remodeling by targeting endothelial cells and refining proinflammatory molecules such as IL-1β, leading to vascular damage and tissue fibrosis (31).

On the other hand, this study also found that the numbers of T-cell CD4 memory resting were lower in patients with SSc-PAH. Although this study regrettably did not carry out further experimental validation in SSc-PAH patients, which is one of its limitations, other studies have demonstrated similar results. Andreas et al. found that the number of T-cell CD4 memory resting was less in the juvenile systemic sclerosis (jSSc) population (33). In addition to adaptive immune responses, many studies have suggested the role of innate immune cells, especially neutrophils, in the pathogenesis of SSc (34–36). The complex clinical and pathological features of SSc complicate the understanding of the function of the immune and vascular systems in the pathogenesis of this severe autoimmune disease.

The infiltration of neutrophils in diseased tissues has been confirmed as an important factor in fibrosis (23). In chronic inflammatory or autoimmune diseases, high levels of inflammatory cytokines drive the formation of neutrophils into neutrophil extracellular traps (NETs) (37–39). Neutrophils function through phagocytosis, respiratory burst, degranulation, reactive oxygen species (ROS) release, formation of NETs, and secretion of cytolytic enzymes, cytokines, and chemokines (37, 40, 41) Furthermore, one study demonstrated the presence of NETs structures in human fibrotic skin biopsies (37). Here, NETs were found in close proximity to α-SMA-positive myofibroblasts in lung and skin biopsy specimens from patients with SSc, suggesting a possible mechanism by which neutrophils drive fibrosis (37).

The number of neutrophils in the peripheral blood may represent the degree of neutrophil infiltration in tissues to a certain extent. In our research, compared with SSc, the numbers of neutrophils in the peripheral blood were significantly increased, which may suggest that there is also differential neutrophil infiltration in SSc-PAH and SSc in vascular tissues. This requires further research to confirm.

Furthermore, in this study, we found another interesting result in **Figure 1C**, the numbers of mast cells and macrophages are small and the difference between the two groups is not statistically significant. However, mast cells are still very essential to the fibrosis of SSc. It has been shown that mast cells in tissues are associated with fibrosis, and in patients with SSc, the density of mast cells in the dermis can reflect the severity of sclerosis (42). The reason for this result may be that the mast cells and macrophages are tissue-infiltrating, and the dataset taken in this study was obtained from PBMCs.

In previous studies, it has been found that in the pathological tissues of SSc, such as the perivascular and peri-adnexal areas between thickened collagen fibers (43), there is a mononuclear cell infiltration, including macrophage infiltration (43). Among them, macrophages can secrete a large number of profibrotic molecules to mediate fibrosis *in situ*. In patients with SSc, the number of monocytes in the peripheral blood was significantly increased compared with healthy subjects (44).

In the present study, a similar conclusion was also observed, that is, a large number of monocytes were detected in the peripheral blood of patients with SSc regardless of the presence of PAH (**Figure 1C**), but there was no statistical difference between the two groups. This may be due to the fact that mononuclear cells in the peripheral blood do not reflect the infiltration in the tissue.

Nonetheless, the differences in the composition of immune cells in the peripheral blood can help us understand changes in circulating immunity during disease development. Also, in certain subsets of cells, the difference in the numbers of immune cells in the peripheral blood can still reveal the tissue changes to some extent. In addition, in the early stages of disease development, it is difficult to obtain histopathology, and the peripheral blood is still a more convenient type of clinical test specimen.

In conclusion, many discoveries on immune alterations between SSc and normal populations have been made, but the immune alterations involving SSc-PAH are still poorly understood. Which hub immune cells, cytokines, and signal transduction pathways are involved in the occurrence of scleroderma pulmonary hypertension is still an urgent problem to be solved.

To further analyze the possible mechanisms responsible for differences in the number of immune cells in the peripheral blood of SSC-PAH and explore the role of inflammation and immune dysregulation in the development and/or progression of IPAH, we analyzed the top 5 most enriched KEGG pathways of those 15 IRGs, which are PD-L1 expression and PD-1 checkpoint pathway in cancer, NF-kappa B signaling pathway, T-cell receptor signaling pathway, and FoxO signaling pathway. The top 5 most enriched GO-molecular function terms are T-cell activation, regulation of T-cell activation, positive regulation of T-cell activation, and positive regulation of leukocyte cell-cell adhesion. These results may indicate that the regulation of T-cell activation is central to the development of SSc.

Many studies have some evidence supporting the critical role of these signaling pathways and immune dysregulation in the pathogenesis of SSc (45). One study showed that stimulation of dendritic cells carrying this variant of SSc with a TLR2 agonist increased macrophage activation, resulting in increased production of interleukin-6 (IL-6) and tumor necrosis factor (46), thus participating in the pathogenesis of SSc. Regulation of the NF-κB pathway is triggered by PRR, cytokine, TCR, and BCR activation (47, 48). Increased expression of NF-κB-regulated cytokines in SSc keratinocytes also suggests that this pathway is activated in SSc pathogenesis (49). Abnormal recruitment of T cells was found in SSc tissues, which may be due to the increased expression of chemokine receptors and the release of chemokines, leading to the recruitment of immune cells to target tissues (45).

Other studies have suggested that many cytokines are also involved in the process of SSc fibrosis. Among them, IL-12, IL-21, and IL-21R are key molecules of fibrosis in the SSc epidermis by promoting the secretion and expression of helper T cells (50–52)

So far, some genes significantly involved in fibrogenesis have been discovered. The current study found the following: c-Src tyrosine kinase (CSK) (53), caveolin1 (CAV1) protein (54), interferon regulatory factor 8 (IRF8), growth factor receptor-binding protein 10 (GRB10), and SRY-box transcription factor 5 (SOX5) (55). They may be related to the fibrosis of SSc, but more research is needed to verify this.

Some research has revealed that hypoxia leads to decreased DDX6 expression, which can induce the translation and secretion of VEGF mRNA, promoting angiogenesis (56). The initial events of SSc persist through endothelial cell and vascular remodeling. VEGF may be a hub gene in SSc-PAH also found in our study. However, we are still unclear why some SSc patients develop secondary PAH. Little research has been done on the early immunological changes and the hub immune-related genes in SSc secondary to PAH. One study has demonstrated downregulated IL-7R expression on CD4 T cells in scleroderma patients with pulmonary hypertension (45), which is consistent with our results.

In order to explore the hub immune-related genes responsible for the changes in the number of immune cells in the peripheral blood during disease development, we screened a total of four hub immune-related genes, respectively, EGFR, LCK, IL-7R, and HDAC1. Through the analysis of gene function, we found that three genes, IL-7R, LCK, and HDAC1, are positive correlated with T-cell CD4 naive and T-cell CD4 memory. At the same time, IL-7R, LCK, and HDAC1 were negative correlated with neutrophils.

According to the previous results, neutrophils have a higher abundance and T-cell CD4 naive and T-cell CD4 memory resting have a lower abundance in SSc-PAH patient PBMC samples. We further explore the relationship between hub genes and these immune cells. Consistent with our predictions, the expressions of IL-7R, LCK, and HDAC1 are positively correlated with the numbers of T-cell CD4 naiveness and T-cell CD4 memory; otherwise, the expressions of IL-7R, LCK, and HDAC1 are negatively correlated with the numbers of neutrophils in the peripheral blood. The result reminds us that these three molecules may be the hub immune-related genes responsible for the reduction of T-cell numbers in the peripheral blood of SSc-PAH. The hub gene analysis in CTD shows that EGFR has high scores in pulmonary arterial hypertension, connective tissue disease, and systemic sclerosis, and may be a key gene involved in the pathogenesis of SSc, especially SSc-PAH. This provides a new idea for our next study on targeted therapy for SSc-PAH.

Thus, in order to verify the expression differences of hub genes IL-7R, LCK, and HDAC1 between SSc and SSc-PAH, we used GSE22356 and GSE33463 as test sets and GSE19617 as the validation sets, which include 21 SSc patients and 15 SSc-PAH patients. The result shows that the mRNA expression levels of the 3 hub genes in the SSc-PAH samples were significantly decreased compared with those in the SSc samples (*p* < 0.05). To determine the significance of IL-7R, LCK, and HDAC1 in the diagnosis of SSc patients, ROC analyses were conducted to explore the sensitivity and specificity of hub genes for SSc diagnosis. This result indicates that the expressions of IL-7R, LCK, and HDAC1 are related to the disease activity of SSc. The low expression of IL-7R, LCK, and HDAC1 detected in the peripheral blood of SSc may indicate the possibility of PAH and hopefully become a biomarker for the early detection of SSc-PAH.

Combined with previous research results, IL-7R has been demonstrated in a microarray cohort as a differentially expressed gene and validated by RT-qPCR in a validation cohort of SSc and SSc-PAH groups. The study confirmed a decreased expression in SSc-PAH (57).

In addition, there is no relevant study on LCK and HDAC1 in SSc and SSc-PAH groups. LCK is a nonreceptor tyrosine-protein kinase that plays an essential role in the selection and maturation of developing T cells in the thymus and in the function of mature T cells. LCK is involved in the T-cell antigen receptor (TCR)-linked signal transduction pathways, as well as the IL-2 receptor-linked signaling pathway that controls the T-cell proliferative response (58).

Also, HDAC1 can catalyze the deacetylation of lysine residues on the N-terminal part of the core histones (H2A, H2B, H3, and H4) (59). HDAC1 plays an important role in transcriptional regulation, cell cycle progression, and developmental events (60) and inhibits the transcriptional activity of NF-kappa-B pathway (61). LCK and HDAC1 may reduce T cells in SSc-PAH PBMCs through the regulation of T-cell activation, which suggest that these three molecules may be involved in the development of SSc-PAH. This requires our follow-up experiments to confirm.

Finally, we explore the microRNA expression related the hub immune genes. Four hub gene expression–related microRNAs were analyzed. Forty-nine target miRNAs of four specifically expressed hub genes and a determined 49 mRNA–miRNA pairs were obtained. This result provides a direction for the following research on the expression and regulation of hub genes and lays the foundation for the study of the mechanism of immune changes in SSc secondary to PAH.

There are still many deficiencies in this study. First, the explored three hub genes and their expressed proteins have not been further validated in PBMCs of SSc-PAH patients. Second, there is a lack of research on the changes of cellular immunity in the peripheral blood and tissues at the same time. Third, this study focused on the cellular immunity changes in SSc and SSc-PAH PBMCs, ignoring healthy controls. These need to be further improved and studied in subsequent experiments.

In the present study, we applied CIBERSORT analysis to find differences in immune cell populations in PBMCs between SSc without PAH and SSc-PAH. Compared with SSc without PAH, SSc-PAH had more neutrophil but fewer T-cell CD4 naive and T-cell CD4 memory resting in the peripheral blood. To further explore the cause and potential molecular events between the two groups, we identified the IL-7R, LCK, and HDAC1 as hub genes involved in the occurrence of SSc-PAH. These three genes could reduce the number of T-cell CD4 naive and T-cell CD4 memory resting in SSc-PAH PBMCs through the regulation of T-cell activation, which suggest that these three molecules may be involved in the development of SSc-PAH. Meanwhile, the low expression of IL-7R, LCK, and HDAC1 detected in the peripheral blood of SSc may indicate the possibility of PAH and hopefully become a biomarker for the early detection of SSc-PAH. Furthermore, whether or not PAH is present, EGFR has a very strong association with SSc and may be a hub gene in the pathogenesis of SSc, providing new ideas for the next targeted therapy.

## Data Availability Statement

The datasets presented in this study can be found in online repositories. The names of the repository/repositories and accession number(s) can be found below: https://www.ncbi.nlm.nih.gov/geo/, GSE22356; https://www.ncbi.nlm.nih.gov/geo/, GSE33463; and https://www.ncbi.nlm.nih.gov/geo/, GSE19617.

## Author Contributions

JT designed and conducted the whole research. JJ applied for the GEO dataset analysis of SSc, software, and visualization. XW analyzed and interpreted the data. ZC and LS revised and finalized the manuscript. All authors contributed to the article and approved the submitted version.

## Funding

This work was supported by the Natural Science Foundation of Zhejiang Province, China (grant number LY20H100002) and Medical Science Research Foundation of Zhejiang Province, China (grant number 2020KY634).

## Conflict of Interest

The authors declare that the research was conducted in the absence of any commercial or financial relationships that could be construed as a potential conflict of interest.

## Publisher’s Note

All claims expressed in this article are solely those of the authors and do not necessarily represent those of their affiliated organizations, or those of the publisher, the editors and the reviewers. Any product that may be evaluated in this article, or claim that may be made by its manufacturer, is not guaranteed or endorsed by the publisher.
