# Supplementary material for: Altered Cellular Immunity and Differentially Expressed Immune-Related Genes in Patients With Systemic Sclerosis–Associated Pulmonary Arterial Hypertension
Source: Front Immunol. 2022 May 18;13:868983. doi: 10.3389/fimmu.2022.868983 (PMC9159786; doi:10.3389/fimmu.2022.868983)
Supplement: Supplementary file 1 [file DataSheet_1.pdf]

Supplementary Material

1.1 Supplementary Figures

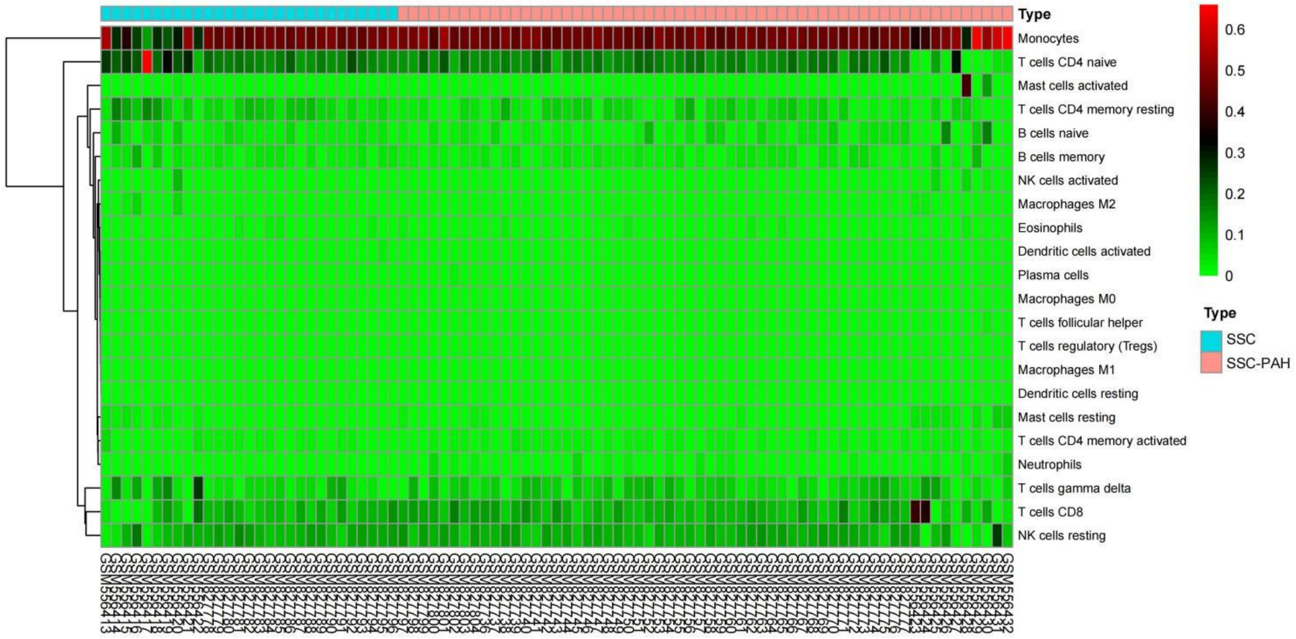

Supplementary Figure 1. The distribution of 22 immune cells in 69 filtered gene matrix. Red indicates higher immune infiltration expression, and green indicates lower expression.

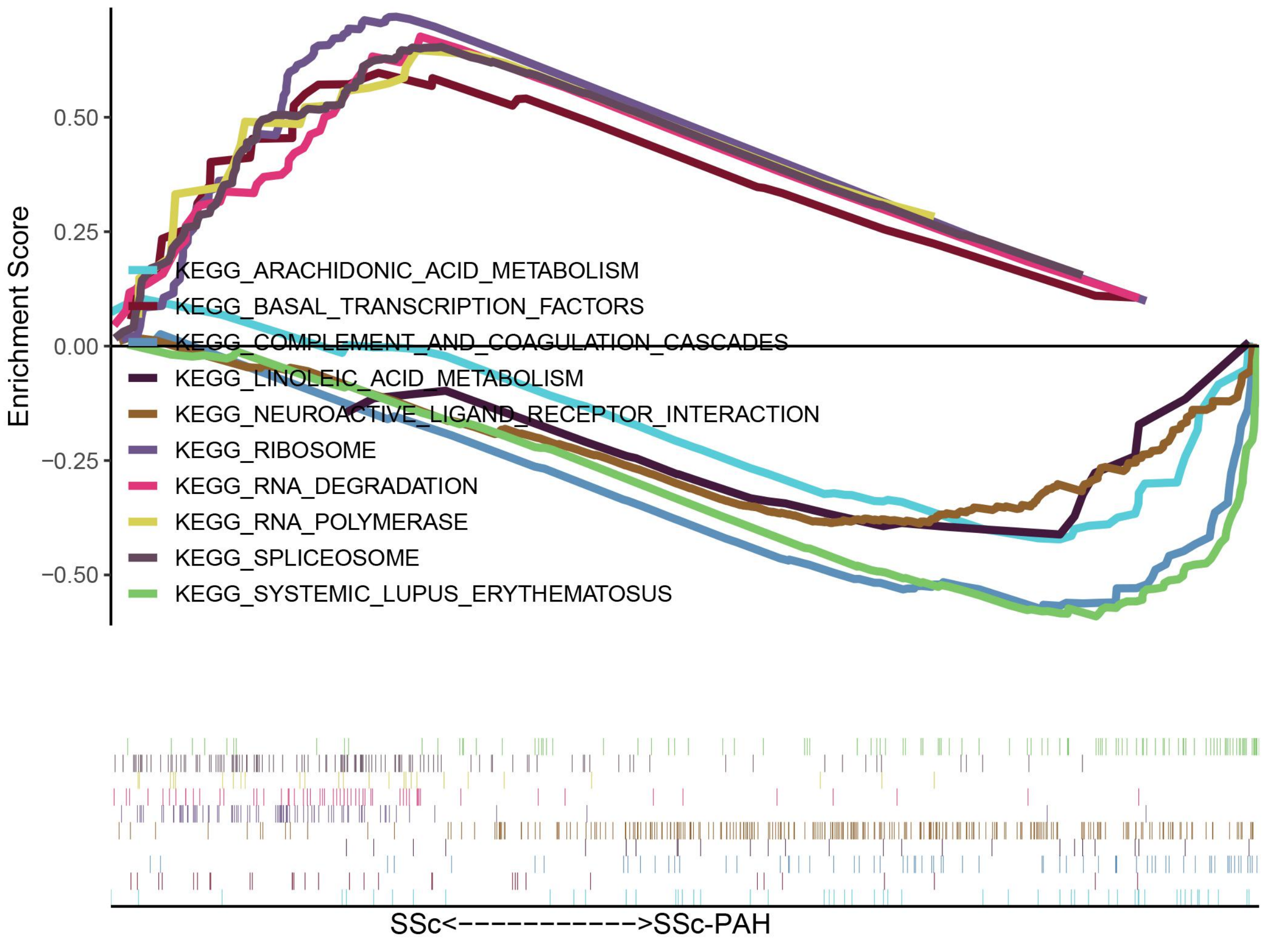

Supplementary Figure 2.The result of GSEA analysis between SSc and SSc-PAH groups.

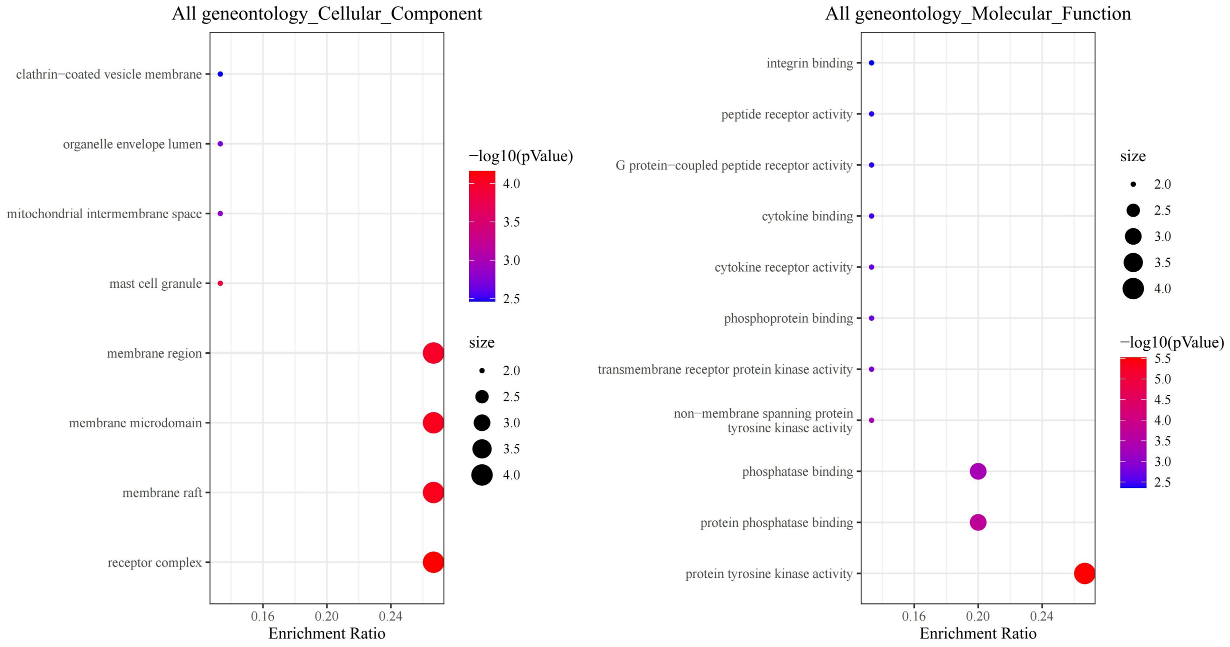

Supplementary Figure 3. The Enriched GO- Cellular Component and Molecular Function terms.

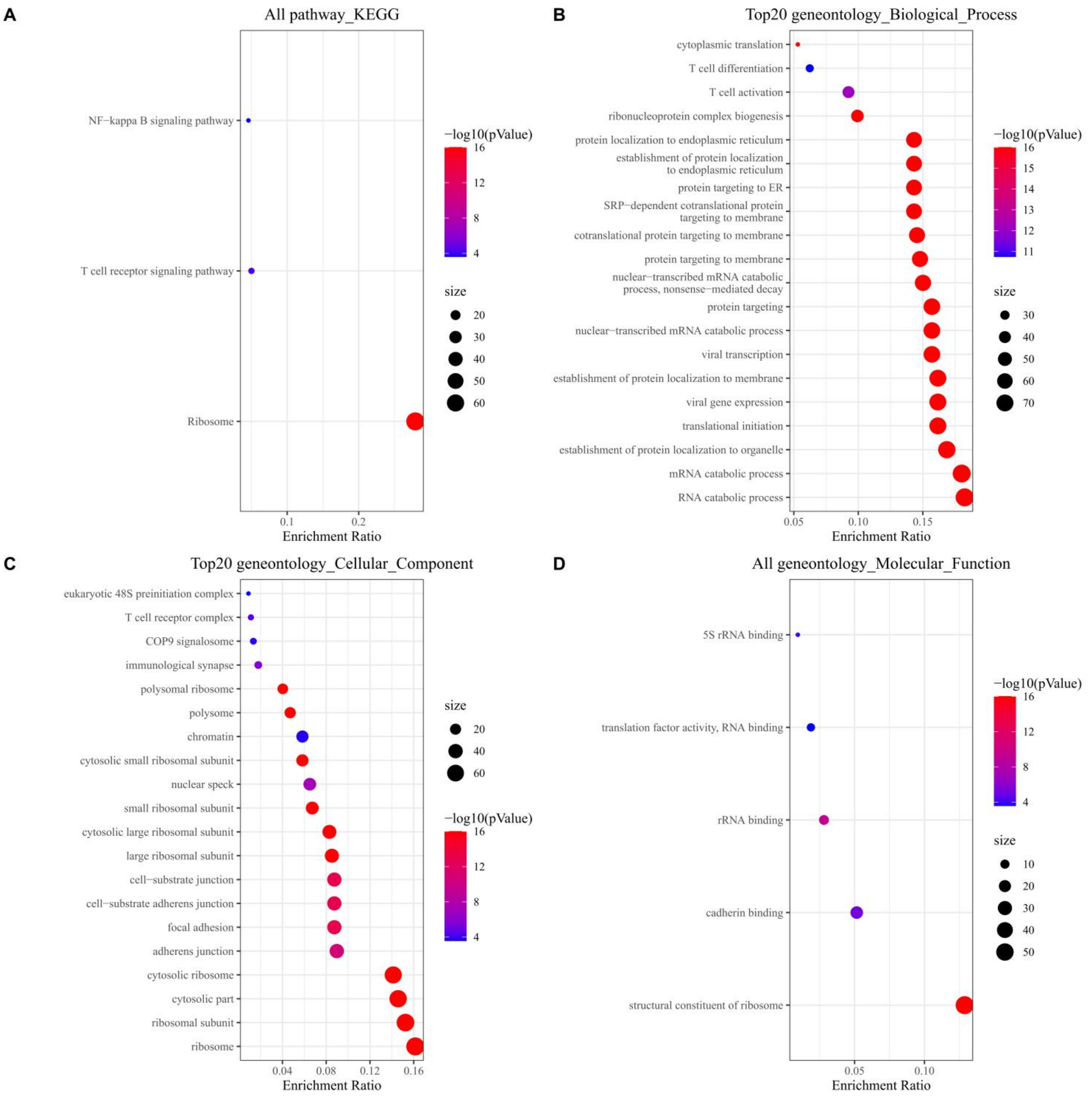

Supplementary Figure 4. GO and KEGG pathway enrichment analysis of the blue module.

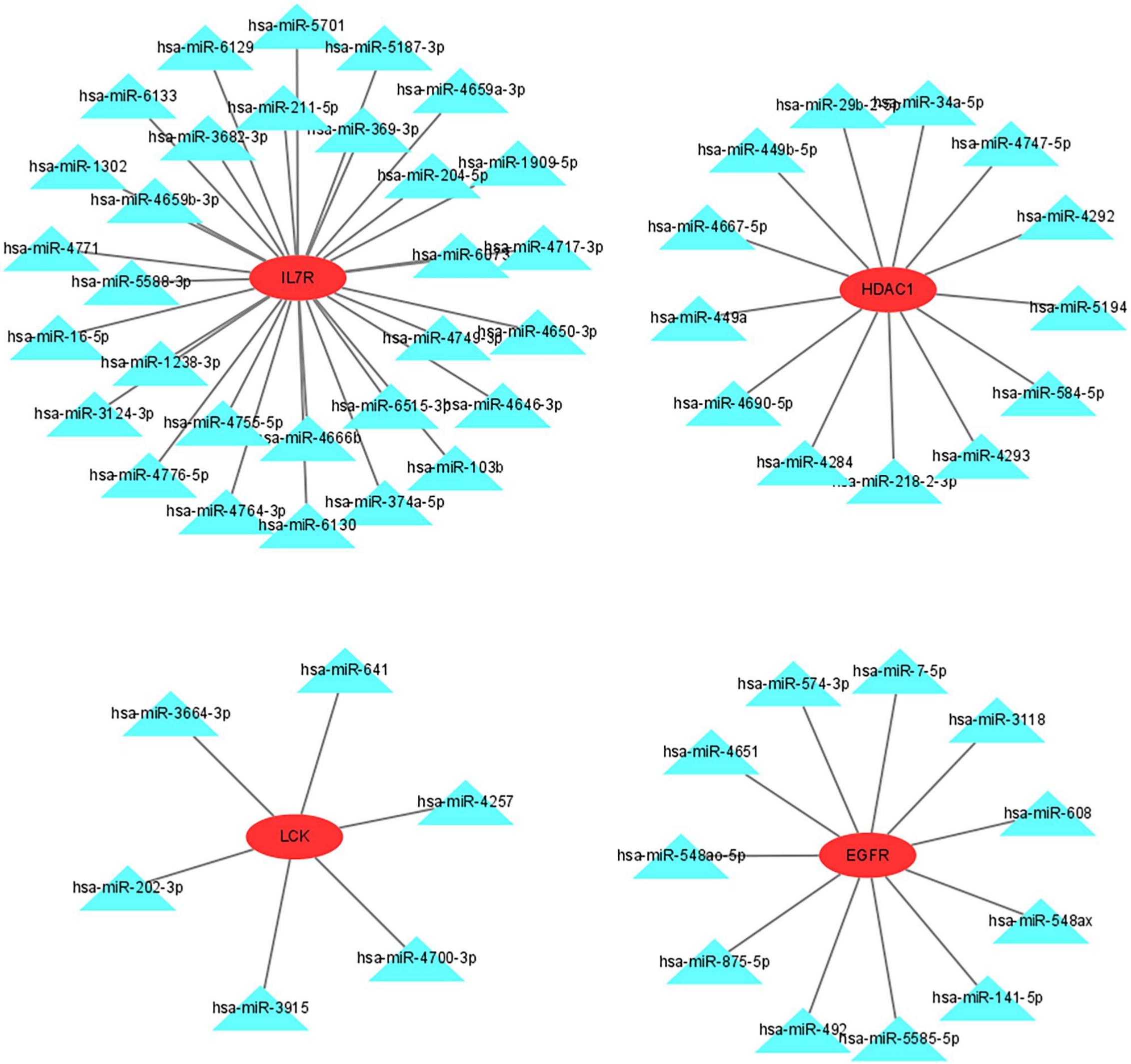

Supplementary Figure 5. The construction of the co-expressed network. Red ovals represent hub genes, and blue triangles represent the target mRNA-miRNA pairs
